# MicroRNA Mediated Regulation of Schwann Cell Migration and Proliferation in Peripheral Nerve Injury

**DOI:** 10.1155/2018/8198365

**Published:** 2018-04-30

**Authors:** Eun Jung Sohn, Hwan Tae Park

**Affiliations:** Peripheral Neuropathy Research Center, Department of Physiology, College of Medicine, Dong-A University, Busan, Republic of Korea

## Abstract

Schwann cells (SCs) contribute to nerve repair following injury; however, the underlying molecular mechanism is poorly understood. MicroRNAs (miRNAs), which are short noncoding RNAs, have been shown to play a role in neuronal disease. In this work, we show that miRNAs regulate the peripheral nerve system by modulating the migration and proliferation of SCs. Thus, miRNAs expressed in peripheral nerves may provide a potential therapeutic target for peripheral nerve injury or repair.

## 1. Introduction

MicroRNAs (miRNAs) are endogenous small noncoding RNAs that are present as RNA-duplex transcripts of approximately 22 nucleotides. miRNAs regulate mRNAs in eukaryotic cells [1]. In the initial step of processing, primary miRNA transcripts, called pri-miRNAs, are generated in the nucleus with a 5′ cap structure and a 3′ poly-A tail [2, 3]. Next, precursor miRNAs containing a 70-nucleotide stem loop precursor miRNA are generated from the pri-miRNA by microprocessor complexes, such as the nuclear Drosha-DiGeorge syndrome critical region gene 8 (DGCR8) enzyme complex [4]. The pre-miRNA is exported to the cytoplasm via binding with the nuclear export factor, exportin 5. Finally, miRNA maturation is mediated by Dicer, a type-III ribonuclease, which allows the miRNA to form a duplex of 22 nucleotides [5]. The mature miRNA is then loaded onto the RNA-induced silencing complex (RISC), which modulates gene expression by binding via imperfect complementarity to the 3′-untranslated region (UTR) of target mRNAs, resulting in translational repression or degradation of the mRNA [6]. miRNAs are involved in diverse biological functions in aging [7, 8], cancer [9, 10], cell development [11, 12], and neuronal disease [13–15].

The peripheral nervous system, which links the brain and body, is made up of the nerves and ganglia outside the brain and spinal cord. Schwann cells (SCs) are important in the development and maintenance of the peripheral nervous system. In adult peripheral nerves, SCs exist in myelinating and nonmyelinating forms [6]. SCs that arise from the neural crest are surrounded by axons and myelinate large-caliber axons in the peripheral nervous system [16]. Schwann cell dysfunction or injury results in demyelinating neuropathies along with loss of motor and sensory abilities [17]. Mutations in SCs contribute to disease by reduction or gain of function of specific genes, such as peripheral myelin protein-22 (PMP22) or myelin protein zero (MPZ) [18]. There is evidence that misfolded PMP22 or MPZ accumulates within the endoplasmic reticulum of myelinating SCs [19].

Krox20 (also termed early growth response gene: EGR2) is a transcription factor that plays a role in myelin formation during promyelination. SCs are important myelin structural components along with myelin binding protein (MBP) and MPZ [20, 21]. Krox20, MBP, and MPZ levels are increased during development of the sciatic nerve while sex determining region Y-box 2 (SOX2), Notch, and Jun act as negative regulators [22–24].

Following injury by nerve transection or crush, SCs undergo a demyelination process and myelin damage called Wallerian degeneration. Macrophages in peripheral nerves move to the site of damage and are activated to clear the damaged myelin or axonal debris [25]. The clearance of myelin leads to axonal regeneration following peripheral nerve injury. Crush injury and nerve trunk transection are common types of experimentally induced nerve injury [26]. Crush injury results in axonal interruption while maintaining the connective sheaths, which is called axonotmesis, while nerve transection results in disruption of axon connection and nerve loss. Degeneration following nerve transection is more acute than following crush injury [26].

## 2. The Proliferation of SCs

SC proliferation is regulated by platelet-derived growth factor [27], transforming growth factor (TGF) [28], laminin [29], Notch [24], and neuregulin 1 (NRG-1) [30]. Notch and neuregulin 1 which are axon-associated signals affect SC proliferation* in vivo*. Notch receptor localizes in SC and Notch ligands exist on axons. Canonical Notch signaling is essential positive regulator of SC proliferation by decreasing the number of SC after Notch inactivation* in vivo* [24]. NRG-1 is also present on axons and its receptors such as ErbB2 and ErbB3 exist in Schwann cells. NRG treatment stimulates SC proliferation* in vitro* [30]. TGF-*β* treatment in Schwann cell enhanced SC proliferation* in vitro* [31]. D'Antonio et al. showed that type II TGF-*β* receptor mutant mice reduced SC DNA synthesis [28]. Laminin which is the major component of the basal lamina also contributes to SC proliferation. Yu et al. showed that laminin-null Schwann cells attenuated phosphatidylinositol 3- (PI3-) kinase activity and proliferation of immature SCs [29]. A recent study showed that mTORC1 (mechanistic target of rapamycin) pathway which is a downstream of growth-factor-stimulated phosphatidylinositol 3′ kinase (PI3K)/AKT pathway enhanced proliferation of immature SC as well as the myelin sheath of differentiated SCs [32]. In addition, SC proliferation is important during Wallerian degeneration by increasing SC number in the distal stump [33].

## 3. The Migration of SCs

After peripheral nerve injury, Schwann cell migrates toward regrowing axons by regulating extracellular matrix (ECM) and ECM substrates such as fibronectin and laminin [34]. Also, extracellular signal-related kinase (ERK) 1/2 and AKT are important players for migration of Schwann cell during nerve degeneration [35]. Chang et al. demonstrated that neuregulin enhanced SC migration through erbB2/3- focal adhesion kinase (FAK) pathway following nerve injury [36]. Bentley and Lee showed that neutrophin receptor p75 mutant mice attenuated SC migration and axon growth [37].

## 4. miRNAs in the Regeneration of Peripheral Nerves

There is evidence that miRNAs modulate peripheral nerve myelination. Absence of Dicer in sciatic nerve SCs in a mouse model impaired myelin formation and reduced mRNA level of KROX20, a key transcriptional activator of myelin genes [38, 39]. Bremer et al. showed that several miRNAs, including miRNA-34a, miRNA-146, miRNA-30a, miRNA-195, miRNA-140, miRNA-27b, and miRNA-204, were upregulated upon myelination in Dicer mutant mice [39]. Gokey et al. showed that 225 miRNAs were expressed during peripheral myelination by microRNA profiling. miRNA-106a, miRNA-20b, miRNA-338, miRNA-92b, miRNA-19b, miRNA-363, miRNA-350, miRNA-17, and miRNA-340 regulated Sox10, which regulates myelin genes [40].

### 4.1. Let-7

The lethal-7 (let-7) gene plays a vital role in carcinogenesis. Moreover, several studies have shown that let-7 miRNAs affect neuronal cell fate and regeneration by regulating nerve growth factor (NGF) expression [41]. Li et al. showed that, following sciatic nerve transection, let-7c, -7d, -7e, -7f, -7i, and miRNA-98 levels in the proximal nerve were increased at 1 day after nerve injury and decreased at 4 and 7 days after nerve injury, followed by a rebound at 14 days. A transwell migration assay showed that transfection with let-7d/miRNA-98 mimics significantly suppressed the migratory ability of SCs compared with the control [41]. NGF has been shown to be regulated by let-7 miRNAs through direct binding to the 3′-UTR of NGF mRNA [41]. Thus, let-7 miRNAs affect SC migration and axon outgrowth during regeneration.

### 4.2. miRNA-1

Expression of miRNA-1 following sciatic nerve crush was drastically decreased at 4, 7, and 14 days after peripheral nerve injury compared with the expression at 0 h, while brain-derived neurotrophic factor (BDNF) expression was enhanced, exhibiting a negative correlation with miRNA-1 [42]. Overexpression of miRNA-1 in primary SCs inhibited the migration and proliferation of SCs. BDNF knockdown by small interfering RNA recapitulated the effects of miR-1 on the proliferation and migration of SCs [42].

### 4.3. miRNA-9

miRNA-9 has been shown to be an important regulator of SC migration [43], which is critical to the regenerative response of SCs to nerve injury. Overexpression of miRNA-9 mimics attenuated SC migration, whereas inhibition of miRNA-9 enhanced migration by directly targeting collagen triple helix repeat containing protein 1 (CTHRC1) [43].

### 4.4. miRNA-148-3p

Qian et al. showed that miRNA 148-3p plays a role in the regeneration of peripheral nerves by regulating SC migration [44]. For instance, overexpression of miRNA 148-3p enhanced the migratory ability of SCs while inhibition of miRNA-148-3p attenuated migration* in vitro*. Furthermore, miRNA-148b-3p enhanced migration via targeting Cullin-associated NEDD8-dissociated protein 1 (Cand1), which negatively regulated the proliferation of lymph node carcinoma cells in the prostate [44].

### 4.5. miRNA-sc8

A transwell migration assay showed that transfection with miRNA-sc8 mimic reduced the migration of SCs, while silencing miRNA-sc8 expression enhanced the proliferation and migration of SCs. In addition, silencing of the epidermal growth factor receptor (EGFR) diminished the positive effects of the miRNA-sc8 inhibitor on SC proliferation and migration [45]. Thus, this evidence suggested that miRNA-sc8 regulates SC proliferation and migration by targeting EGFR.

### 4.6. miRNA-210

Zhang et al. showed that miRNA-210 modulates peripheral nerve regeneration by enhancing the migration and proliferation of SCs [46]. miRNA-210 levels increased until 14 days after sciatic nerve injury. In addition, overexpression of miRNA-210 enhanced the migration and proliferation of SCs. Moreover, miRNA-210 transfection increased the expression of growth associated protein 43 (GAP43) [46]. Thus, these findings suggested that upregulation of miRNA-210 following sciatic nerve injury may be important for peripheral nerve regeneration.

### 4.7. miRNA-221/222

Yu et al. performed Agilent miRNA microarray analysis to examine the expression profile of miRNAs in the proximal stump of nerves following sciatic nerve transection [47] and showed that, among 77 miRNAs, miRNA-21, miRNA-31, miRNA-221, miRNA-222, and miRNA-132 levels were enhanced at 1 day after sciatic nerve injury. In situ hybridization revealed that miRNA-221/222 was highly expressed at 4 days after sciatic nerve injury. A transwell migration assay demonstrated that miRNA-221/222 overexpression increased the migratory ability of primary SCs, while miRNA221/22 inhibitor impaired SC migration. miRNA-221/222 modulates longevity assurance homolog 2 (LASS2) in SCs by binding directly to the 3′-UTR of LASS2 mRNA. Thus, sciatic nerve injury enhanced SC migration by regulating miRNA-221/222, which targets LASS2 [47].

### 4.8. miRNA-sc3

miRNA-sc3 has been shown to be highly expressed in the injured nerve following sciatic nerve transection [48]. Increased expression of miRNA-sc3 promoted the proliferation and migration of primary SCs, while silencing of miRNA-sc3 attenuated proliferation and migration. miRNA-sc3 directly targeted astrotactin 1* (Astn1)* and led to translational suppression of Astn1. Thus, these data suggested that miRNA-sc3 modulates the migration and proliferation of primary SCs via Astn1 [48].

### 4.9. miRNA-132

Yao et al. showed that miRNA-132 expression was increased following sciatic nerve injury. Furthermore, miRNA-132 enhanced the migration of primary SCs. miRNA-132 also enhanced SC migration in hypoxic conditions, while miRNA-132 inhibitor suppressed migration [49]. This study showed that miRNA-132 modulates SC migration by suppressing protein kinase AMP-activated noncatalytic subunit gamma 3 (PRKAG3) expression by binding the 3′-UTR of its mRNA [49].

### 4.10. miRNA-182

Yu et al. demonstrated that miRNA-182 was highly expressed following sciatic nerve resection by quantitative reverse transcription polymerase chain reaction, in situ hybridization, and miRNA array analysis [50]. Transwell migration and proliferation assays showed that miRNA-182 overexpression significantly attenuated the migration of primary SCs. In addition, miRNA-182 repressed primary SC proliferation and migration through fibroblast growth factor 9 and neurotrimin, implicating miRNAs in peripheral nerve repair [50].

## 5. MicroRNAs in Peripheral Neuropathy

Damage or dysfunction of the nervous system causes neuropathic pain [51]. While the number of patients experiencing neuropathic pain has increased worldwide [51], the molecular pathogenesis has yet to be elucidated. Therefore, it will be important to understand the molecular mechanism of neuropathic pain and develop novel therapeutic targets. Evidence indicates that miRNAs are involved in neuropathic pain. miRNA-203 attenuated the development of neuropathic pain via regulation of Rap1a expression in neuronal PC12 cells [52]. Expression of miRNA-183 also resulted in suppression of neuropathic pain by repressing the mTOR/VEGF signaling pathway [53]. miRNA-93 was found to attenuate neuropathic pain via modulation of signal transducer and activator of transcription 3 (STAT3) [54]. Recent studies have shown that miRNAs affect peripheral neuropathy.

### 5.1. miRNA-146a

miRNA-146a plays a role in diabetic peripheral neuropathy [55, 56]. miRNA-146a in sciatic nerve tissue was attenuated in mice with type 2 diabetes and miRNA-146a levels in plasma and sciatic nerve tissue were enhanced following administration of miRNA-146a mimics in diabetic mice. Additionally, miRNA-146a mimics enhanced the axonal diameter and myelin thickness of sciatic nerves [56].

## 6. The Role of microRNAs in SC Myelination 

A recent study showed that Dicer, which is a player for miRNA biogenesis, is important for SC myelination. For instance, Dicer deficient SCs diminished their ability to myelinate and microarray analysis of miRNA from Dicer deficient nerves showed that 109 miRNAs were significantly up- or downregulated. miRNA-1224, miRNA-9, and miRNA-455 were reduced upon myelination and downregulated following Dicer mutant from SCs [39]. Yun et al. showed that miRNA 138 showed a developmental increase between P2 and P21 while it repressed Ccnd1, Sox2, or c-jun which are negative regulators of myelination implying that miRNA138 might be essential for SC differentiation [57]. Also, the expression of miRNA-193, miRNA-222, miRNA-129, miRNA-145, and miR-29a was significantly downregulated in inhibition of Dicer [58]. Gökbuget et al. showed that let-7 miRNAs were significantly upregulated during myelination and inversely correlated to the level of lin28 homolog B (Lin28B) which is a microRNA regulator. Lin28B expression leads to impaired onset of SC myelination and let-7 miRNAs enhanced expression of Krox 20, which is a key transcription factor for myelination, via suppression of Notch signaling [59]. These results imply that the Lin28/let-7 regulatory axis may be an important player during remyelination.

## 7. MicroRNAs in Demyelination Diseases

Though several studies reported that miRNAs expressed from active and inactive demyelinating lesions [60] and interleukin 17- (IL-17-) producing T helper cells from peripheral blood cells [61–63] from patients with multiple sclerosis which is a chronic disease of the central nervous system, a few studies for miRNA expression in demyelination disease of peripheral nervous system have been studied. Guillain–Barré syndrome (GBS) which is an acute inflammatory demyelinating polyneuropathy affects the peripheral nervous system [64]. Lv et al. showed that has-miR-4717-5p and has-miR-642b-5p were upregulated in the GBS patients [65]. Also, low level of miRNA155 was expressed in peripheral blood mononuclear cells from GBS patients and silencing of miR155 enhanced the production of Th1-type cytokines such as TNF-*α*, IL-1*β*, IFN-*γ*, and IL-12* in vitro* [66]. To identify miRNAs in demyelinating diseases may be important for developing new biomarkers for demyelinating diseases.

## 8. Conclusion

**Figure 1:**
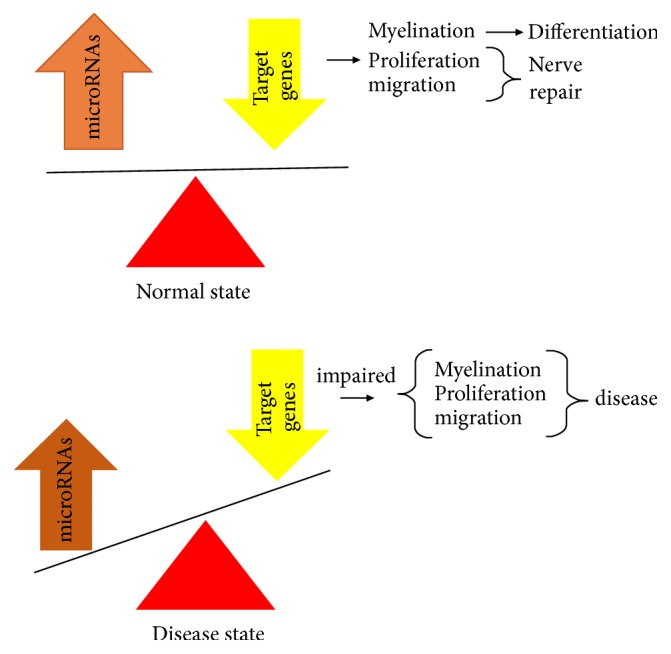
Alternation of microRNA expression and their target contribute to impaired onset of SC differentiation or nerve regeneration.

MiRNAs affect proliferation, migration, and myelination for SCs by regulating their targets in normal state. As shown in Figure 1, alterations in miRNA expression and their target may lead to dysfunction for SC differentiation or nerve regeneration. Therefore, it is essential to identify miRNA expression during development and in disease states which may provide important information for therapeutic and diagnostic use. Also, due to stable expression of miRNA during the tissue sampling [67], miRNA is suggested as a useful tool for biomarker.

**Figure 2:**
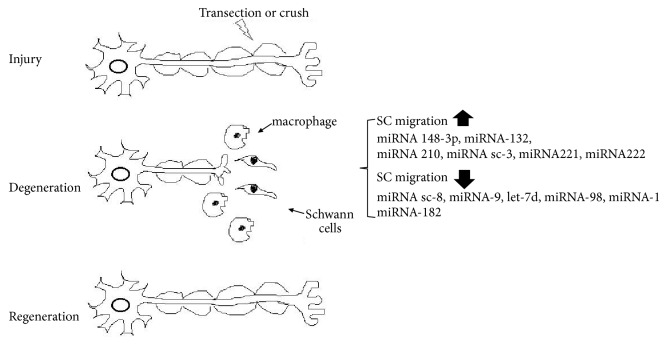
MicroRNAs regulate SC migration during nerve regeneration: miRNA 148-3p, miRNA-132, miRNA 210, miRNA sc-3, miRNA221, and miRNA222 increased the ability of SC migration while miRNA sc-8, miRNA-9, let-7d, miRNA-98, and miRNA-1miRNA-182 downregulated the ability of SC migration.

Taken together, these studies demonstrate the importance of miRNAs in peripheral nerves. SC migration plays an important role in peripheral nerve regeneration following injury. As shown in Figure 2, miRNA-148-3p, miRNA-210, miRNA221/222, and miRNA-sc3 enhanced the migration of SCs while miRNA-sc8, miRNA-9, let-7d, miRNA98, miRNA-1, and miRNA-98 attenuated migration. The miRNAs in these studies contributed to peripheral nerve regeneration. Therefore, the regulation of miRNAs in peripheral nerves may provide a potential therapeutic target for peripheral nerve injury or neuropathy.
